# Australian and United States Consumer Acceptance of Beef Brisket Cooked Using the Low and Slow Barbeque Method

**DOI:** 10.3390/foods13193049

**Published:** 2024-09-25

**Authors:** Jarrod Lees, Nicholas Hardcastle, Justin Johnston, Rohen Wong, Holly Cuthbertson, Garth Tarr, Andrea Garmyn, Markus Miller, Rod Polkinghorne, Peter McGilchrist

**Affiliations:** 1 School of Environmental and Rural Science, University of New England, Armidale, NSW 2350, Australia; j.lees@uq.edu.au (J.L.); rod.polkinghorne@gmail.com (R.P.); 2 School of Agriculture and Food Sustainability, The University of Queensland, Gatton, QLD 4343, Australia; 3 Department of Animal and Food Sciences, Texas Tech University, Lubbock, TX 79409, USA; nick_hardcastle@cargill.com (N.H.); jejohnston@hormel.com (J.J.); garmynan@msu.edu (A.G.); mfmrraider@aol.com (M.M.); 4 School of Mathematics and Statistics, University of Sydney, Sydney, NSW 2006, Australia; rohen.wong@sydney.edu.au (R.W.); garth.tarr@sydney.edu.au (G.T.); 5 Birkenwood International Pty Ltd., 45 Church St., Hawthorn, VIC 3122, Australia; holly.cuthbertson1@gmail.com; 6 Department of Food Science and Human Nutrition, Michigan State University, East Lansing, MI 48824, USA; 7 Department of Animal Science, Michigan State University, East Lansing, MI 48824, USA

**Keywords:** consumer sensory, *M. pectoralis profundus*, *M. pectoralis superficialis*, Meat Standards Australia, smoking, tenderness, juiciness, flavor, overall liking

## Abstract

Meat Standards Australia (MSA) sensory protocols have been effectively utilized in beef for international consumers employing several cooking methods. Our objective was to compare the consumer response of Australian and American consumers to paired beef brisket samples utilizing a newly developed low and slow cooking method. Briskets were collected from Australian carcasses with diverse eating quality. Half of the briskets (n = 24) were retained in Australia and their pair was exported to Texas for consumer sensory testing. Naïve consumers (Australia; n = 240) and familiar consumers (USA; n = 240) evaluated paired barbequed briskets for tenderness, juiciness, flavor liking, and overall liking from 0 to 100 using a visual analogue scale, and a weighted composite meat quality score was later calculated. Australian consumers scored briskets lower for tenderness (−4.84 ± 1.70 points) and juiciness (−4.44 ± 1.55 points) and higher for flavor liking (3.48 ± 1.58 points); however, there was no difference between the countries for overall liking (*p* = 0.75) and combined meat quality score (*p* = 0.88). Differences between Australian and US consumers’ evaluations indicate that there is an impact of cultural background, potentially driven by Australia’s naivety to the low and slow barbeque cooking method.

## 1. Introduction

The Meat Standards Australia (MSA) eating quality grading program delivers a cut by cook eating quality prediction for beef from a group of quantifiable inputs [1,2]. The MSA program is continually evolving, with ongoing testing, using untrained consumers, for new and existing cooking methods for both domestic and international markets [2]. The untrained consumer sensory panels score beef samples for tenderness, juiciness, liking of flavor, and overall liking. To date, beef brisket has not been evaluated within the MSA program using a low and slow cooking method, such as the Texas barbeque technique. Smoking beef is a long-standing practice in the USA and while the trend of smoking meats is generating considerable interest in Australia, it remains in its infancy.

Little research has been conducted on beef eating quality using the low and slow cooking method for items like brisket or short ribs that possess high collagen content [3]. Numerous researchers have demonstrated the toughness of beef brisket through sensory evaluation or shear force testing [3,4,5], but this historic research utilized rapid dry heat cookery. Researchers have begun to assess eating quality of beef brisket prepared using the “low and slow” cooking method, otherwise known as Texas-style barbeque. Harris et al. [6] examined the effect of postmortem aging on brisket eating quality and examined differences in the point (superficial) and flat (deep) portions. Fletcher et al. [7] utilized similar protocols as the current study to investigate brisket eating quality, focusing on the interaction of USDA quality grade and muscle portion.

Studies on the palatability of new cooking methods for the MSA program have garnered differing results when offering consumers non-traditional or novel cooking methods. Previous evaluations of consumer acceptance of non-traditional cooking methods established that Japanese consumers tended to score paired grilled beef samples more harshly for tenderness, juiciness, liking of flavor, and overall liking when compared to Australia consumers [8]. Likewise, Korean consumers tended to score paired beef samples lower than Australian consumers [9]. This suggests that there is potential for consumers to apply divergent palatability scores when presented with culturally novel cooking methods. In addition, Australia produces high volumes of beef each year, exporting a large proportion to countries such as the United States. Therefore, it is critical to compare the sensory response of consumers in key export markets to ensure the MSA prediction model is meeting expectations wherever Australian beef is consumed. To date, no cross-country comparisons have been conducted on brisket eating quality utilizing this relatively novel, at least for research purposes, cooking technique.

Our aim was to investigate the consumer response to the low and slow barbeque cooking method for inclusion into the MSA program utilizing both Australian (naïve) and American consumers from the USA, who are more familiar with this cooking method and the presentation of beef samples in this form. It was hypothesized that there would be no difference in the consumer eating quality scores of briskets cooked using a low and slow cooking method by naïve Australian and familiar USA consumers given the similarity in their cultural backgrounds.

## 2. Materials and Methods

All sensory data presented here were collected with the approval of the University of New England Human Ethics committee (HE17-253) and the Texas Tech University Institutional Review Board (IRB2017-598).

### 2.1. Brisket Collection

Briskets were harvested from twenty-four (24) carcasses of high-, medium-, and low-quality, as determined using the MSA eating quality grading model, from two abattoirs: one in southern Australia and the other in northern Australia. The carcasses represented a mixture of sexes (18 males and 6 females), feed (half on accredited grain (AUS-MEAT Limited, 2018, Murarrie, QLD, Australia) and the other half on grass) and hormonal growth promotant (HGP) treatment (10 were HGP-treated). From each carcass, a point-end (PE) brisket (Handbook of Australian Meat (HAM) #2332) was collected from both sides (left/right) during boning, giving n = 48 briskets. All briskets were frozen (−20 °C) at 3 days postmortem and remained frozen during transport.

Briskets from each carcass were paired between Australian and American consumers with left and right sides rotated between countries: brisket from one side was prepared and tasted at the University of New England, Armidale, Australia; brisket from the other side was prepared and tasted at Texas Tech University, Lubbock, United States of America. Brisket preparation in both countries followed the same protocol (see below). Treatment conditions within each brisket pair were also identical, so the only varying factor was the country of sensory evaluation.

### 2.2. Selection of Briskets for Consumer Sensory Testing

Briskets were selected for inclusion in consumer sensory sessions (n = 8) using a 6 × 6 Latin Square design. Each sensory session included PE briskets with high-, medium-, and low-quality carcasses, as determined by the MSA eating quality grading model. Each sensory session required sufficient product to ensure that 60 consumers received six individual portions of meat, excluding a common link sample at the beginning of the session. The link samples were procured from PE briskets unrelated to the trial from ungraded commodity beef for USA picks or grain-fed commodity briskets for Australian picks.

### 2.3. Preparation of Briskets for Consumer Sensory Testing

Briskets were thawed at 2–4 °C from 72 h prior to cooking. From 14 h prior to cooking subcutaneous fat was trimmed from each PE brisket to approximately 6 mm. This occurred in the same refrigerated room where thawing took place. Endomysium was removed from the medial side of the *M. pectoralis profundus*. Each brisket was weighed prior to cooking. Briskets were seasoned with 1:1 by volume salt and pepper at 0.05% of the raw weight. Each brisket was pinned with an individually identifiable stainless steel ovenproof tag prior to cooking and the tag ID was recorded against the brisket ID. Once seasoned, briskets were returned to storage at 1 °C and cooked the following day.

Briskets were cooked using Green Mountain Grill and Jim Bowie pellet smokers using Green Mountain Grills proprietary “Gold” hardwood blend (Green Mountain Grills LLC, Reno, NV, USA). The smoker temperature was set to 120 °C. Once at temperature, briskets were placed fat side down on the smokers and the time and temperature of each brisket was recorded.

Internal brisket temperature was monitored by placing the proprietary smoker temperature probe in the smallest brisket within the smoker. Additionally, temperature was monitored on all briskets using a handheld meat thermometer (Thermapen Mk4, ThermoWorks, American Fork, UT, USA). When the internal temperature reached 65.6 °C briskets were removed and wrapped in heavy duty aluminum foil and returned to the smoker in the same orientation with time and temperature recorded. When the internal temperature reached 93.3 °C, briskets were removed, retained in the foil wrapping and placed in an insulated holding box with time and temperature recorded. Briskets rested for a minimum of 30 min prior to portioning. Ninety (90) minutes prior to the planned consumer sensory panel start time, briskets were removed from storage, unwrapped, weighed, and portioned for the sensory samples.

The PE briskets were separated into their two muscles (*M. pectoralis profundus* and *M. pectoralis superficialis*) prior to portioning. Each *M. pectoralis profundus* muscle was dissected into a navel and point position (PEB056); *M. pectoralis superficialis* was dissected into a dorsal (PEB057D) and ventral (PEB057V) position. Initial analysis (below) suggested no position difference within *M. pectoralis profundus* and will be referred to as PEB056. Each position was then labelled with an eating quality sample reference number, and assigned a serve style (sliced, pulled, or chopped), prior to portioning into sensory samples. Intermuscular fat and epimysium were removed prior to portioning into sensory samples.

Preparation styles included sliced (6 mm × 70 mm × 40 mm), pulled (70 mm × 10 mm), or chopped (10 mm × 10 mm × 10 mm). Sliced samples were prepared using a cutting guide set to 6 mm, cutting perpendicular to the grain. Chopped samples were prepared by cutting the samples into 10 mm cubes. Pulled samples were prepared by separating the muscle fibers in a sample and cutting them to length with the muscle fibers (70 mm), after which the server selected a 10 mm diameter subset for service to the consumer. Serving style was rotated between different positions on each muscle.

### 2.4. Consumer Sensory Testing

The samples were served according to the MSA sensory testing protocols for a beef roast [10]. Each sample was tested and scored by 10 untrained consumers. In brief, data were collected on tenderness, juiciness, flavor liking, overall liking using a 100 mm visual analogue scale ranging from 0–100, from a total of 240 consumers in each country (480 consumers in total). Panelists were eligible to participate provided they met the following criteria: 18 years of age or older, regularly ate beef, and were willing to consume beef samples cooked to a medium (hot, pink center) degree of doneness. A summary of the participants’ demographic information can be found in Table 1. The visual analogue scale was anchored by descriptions which were not tender/very tender, not juicy/very juicy, and dislike extremely/like extremely for both liking of flavor and overall liking scores, i.e., 0 indicated a not tender sample and 100 was used to describe a very tender sample. A composite meat quality score (MQ4) was then calculated from the outcomes of these four attributes using a discriminant analysis [10]. The weightings for each trait were 0.3 for tenderness, 0.3 for liking of flavor, 0.3 for overall liking, and 0.1 for juiciness, as per the current version of the MSA model [11].

### 2.5. Statistical Analysis

The statistical computing language R (Version 4.4.0) and the tidyverse suite of packages were used for data cleaning, transformation, and visualization [12,13]. The lme4 package [14] was used to fit linear mixed effects models, and the emmeans package [15] was used for post hoc comparisons.

Raw consumer sensory scores (tenderness, juiciness, flavor, overall liking and MQ4) were averaged by sample to represent aggregate eating quality. This gave 184 aggregated scores for each sensory trait. Linear mixed effects models were then built for each sensory trait, with sex, feed type (grass or grain), HGP status (yes or no), rib fat thickness, log of carcass weight, log of ossification score, log of MSA marbling, ultimate pH, loin temperature at grading, cooking loss percentage, pre-cook weight, cooking time, position in muscle, serve method (chopped, pulled or sliced), country tasted (USA or AUS), the interactions between position in muscle and country tasted, position in muscle and serve method, and serve method and country tasted, as fixed effects in the model. Each model utilized the carcass ID as the random effect to capture the within-carcass similarity of briskets. Similarly, primal ID was used to account for the similarity of briskets sensory samples from within the same primal.

Initial analysis suggested that within *M. pectoralis superficialis*, the ventral position scored higher sensory than the dorsal, most notably in tenderness and MQ4. No position difference was suggested within *M. pectoralis profundus*. To account for this nested interaction, we modelled *M. pectoralis profundus* as a whole and allowed for a position effect (dorsal or ventral) in the *M. pectoralis superficialis* (denoted as position in muscle).

Sex, feed, and HGP treatment were included in the modelling as carcass-level covariates. Additional carcass-level covariates are summarized in Table 2. Note that during modelling, carcass weight, ossification score, and MSA marbling score were log-transformed to be on similar scales as other covariates.

Models also included primal-level covariates, summarized in Table 3. Cold weight is weight of the primal prior to cooking (kg). Cooking loss is calculated as (weight after cooking/cold weight) × 100. Cooking time is the amount of time (in hours) taken for the primal to reach 93.3 °C, as per preparation methods above.

Two-way interactions between country tasted, serve, and position in muscle were included in the modelling, being of research interest. For each model, backwards stepwise selection (using the F-test) was used to retain significant (at level 0.05) fixed effects. Where country tasted or serve were dropped, these were re-included to allow post hoc analysis.

## 3. Results

Table 4 shows estimated model coefficients for the final sensory models obtained through backwards stepwise selection.

Position in muscle and serve were found to be significant for all sensory traits (Table 5). Country tested was found to be significant for juiciness, tenderness, and flavor liking but not for overall liking and MQ4. Two-way interactions between country tasted, serve, and position in muscle were dropped during variable selection for every sensory trait. For reference, they were fitted back into the final models for each sensory trait (Table 5); F-test *p*-values show they are not significant. Thus, the next sections analyze the marginal effects of country tested, position in muscle, and serve.

### 3.1. Country Effect

**Figure 1:**
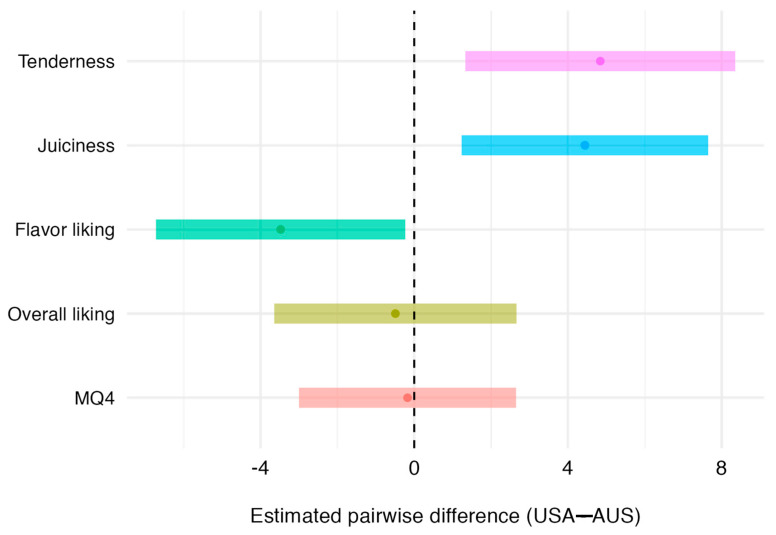
The 95% confidence intervals for the contrast (USA–AUS), for each sensory score. Dots represent the mean differences.

Table 6 summarizes the difference between Australian and USA consumers for tenderness, juiciness, flavor liking, overall liking, and MQ4. USA consumers scored tenderness and juiciness an estimated 4.84 (*p* < 0.001) and 4.44 (*p* < 0.01) points higher. Australian consumers scored flavor liking 3.48 (*p* = 0.037) points higher. No significant country difference was found for overall liking and MQ4. The 95% confidence intervals for the contrast between countries (USA–AUS) for each sensory trait corroborate these results (Figure 1).

### 3.2. Position in Muscle

**Figure 2:**
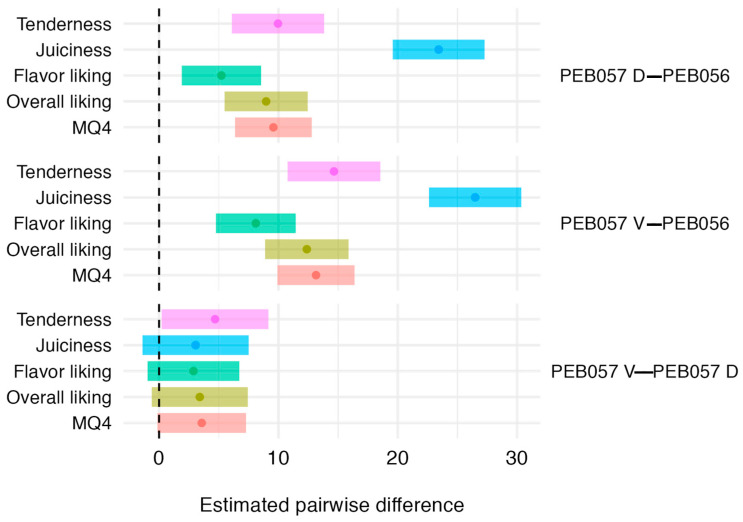
The 95% confidence intervals for pairwise sensory differences between positions in muscles. Dots represent the mean differences. Dashed line represents 0 or no difference.

Figure 2 shows the 95% confidence intervals for the pairwise differences in sensory traits between PEB056, PEB057 D, and PEB057 V. PEB057 D and PEB057 V scored significantly higher in all sensory traits than PEB056. For example, PEB057 D scored an estimated 9.96 (*p* < 0.001), 5.22 (*p* < 0.001), 8.97 (*p* < 0.001), and 9.58 (*p* < 0.001) points higher in tenderness, flavor liking, overall liking, and MQ4 than PEB056. Juiciness scores differed the most: PEB057 D and PEB057 V scored an estimated 23.43 (*p* < 0.001) and 26.48 (*p* < 0.001) points higher than PEB056.

Between the dorsal and ventral positions of PEB057, the only significant difference was for tenderness, with the ventral position scoring higher (*p* = 0.037). While not statistically significant, the other pairwise differences strongly suggest that the ventral position is consistently superior across all sensory traits by 2 to 4 points.

### 3.3. Serve

**Figure 3:**
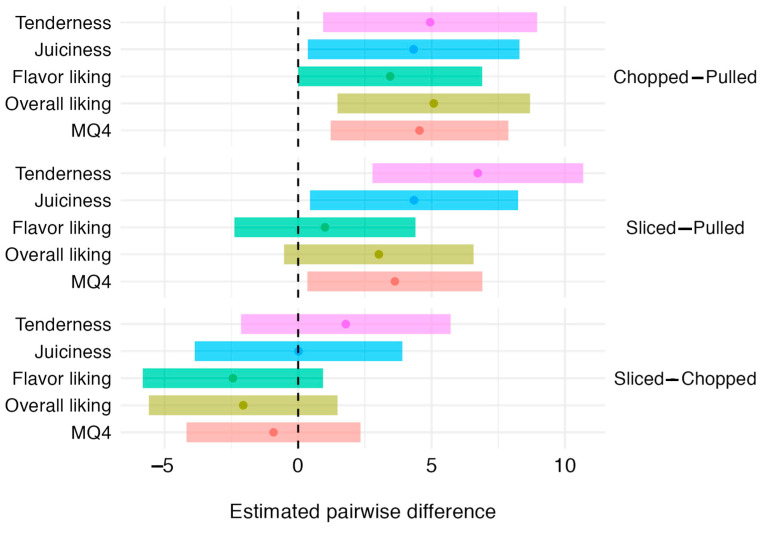
The 95% confidence intervals for the pairwise difference in sensory between serves. Dots represent the mean differences. Dashed line represents 0 or no difference.

Figure 3 shows 95% confidence intervals for pairwise differences in sensory traits, between each serve. No significant difference was found between chopped and sliced serve for all sensory traits. However, the chopped and sliced serves scored significantly higher sensory scores than the pulled serve. The chopped serve scored significantly higher in all sensory traits, with close to a 5-point increase. The sliced serve scored significantly higher than the pulled serve in tenderness, juiciness, and MQ4—the most noticeable improvement being the estimated 6.74 (*p* < 0.001) point-increase in tenderness.

## 4. Discussion

We hypothesized there would be no differences between Australian and American consumers in the eating quality scores of briskets given the similarity in their cultural backgrounds. However, Australian and USA consumer sensory scores differed for tenderness, juiciness, and flavor liking, partially rejecting the hypothesis, since overall liking and the composite MQ4 score was similar between countries. The USA consumer sensory scores were greater for tenderness and juiciness (*p* < 0.01) but lower for the liking of flavor (*p* < 0.05) across all muscle positions and serve methods compared to the Australian consumers. These trait level differences cancelled each other out and were hidden when considering the aggregate overall or MQ4 scores, where there was no significant difference between Australian and USA consumers. A recent review conducted by Bonny et al. [16] outlined that the sensory responses to the evaluation of beef quality from consumers from various cultures and countries are similar. Furthermore, demographic effects from 4140 consumers had a minor impact on consumer sensory scores of sheep meat [17]. While it is difficult to determine why there was a difference in tenderness and juiciness scores between USA and Australian consumers, a study by Polkinghorne et al. [8] found that Japanese consumers weighted the juiciness of grilled beef samples higher than Australian consumers. Polkinghorne et al. [8] suggested that the difference in Japanese and Australian consumers may have been a result of the presentation of beef in a manner the Japanese are not accustomed (i.e., grill). However, this phenomenon was not replicated in a study conducted by Thompson et al. [9], with Korean consumers not necessarily discriminating against the grill method. However, these studies in conjunction with the results from the current study demonstrate the potential effect of a naïve population exposed to a novel cooking method. The differences noted in the current study may be reflective of USA consumers placing more importance on juiciness, and possibly tenderness, in smoked brisket. It could also be indicative that the briskets used in this experiment were more tender and juicier than what they generally experienced in their own country.

The difference in flavor between the two consumer groups may be explained by the relative exposure that each country has to this cooking method. Australians, as naïve consumers to smoked meats, may like the flavor of this novel cooking method more, or USA consumers may find this meat lacking in the flavors associated with additional seasoning that would normally be applied to the meat prior to cooking. A light salt and pepper rub was applied at 0.05% of the brisket weight, far less than the amount of rub applied in a commercial setting, adding to the low flavor scores for American consumers. A salt-and-pepper-only rub also has fewer spices and less complexity of flavor than what is traditionally served. Vázquez-Araújo et al. [18], using a trained taste panel, concluded that a high initial flavor impact was important to USA consumers, as compared to Argentinian and Spanish consumers, and the addition of salt improved sensory outcomes. Similarly, a study on the sensory evaluation of pork smoked with different woods outlined that the results may have been distorted by consumer expectations for a Kansas City-style flavor, which includes a heavy seasoning that was not present [19]. Alternatively, or additionally, USA consumers may have also been expecting a more flavorsome smoke flavor from some of the more typical wood smokes used for red meat, like mesquite and hickory. The use of a less intense wood smoke for this experiment may have also added to the low flavor scores for American consumers.

The serve method influenced the consumer sensory outcome for the PEB056, PEB057 D, and PEB057 V, with pulled samples producing the lowest scores. This was likely due to the effect of fiber length on palatability. Hwang et al. [20], utilizing the same sensory protocols as the current study, outlined the link between the tenderstretch method of hanging the carcass and tenderness in *M. triceps brachii*, *M. longissimus dorsi*, and *M. semimembranosus*, but noted that the cooking method had an impact on the tenderness outcome. Specifically, consumers were served a 25.4 mm thick grill sample and a 4 mm thick Korean BBQ sample, rating the tenderness of the Korean BBQ sample higher than the grill for *M. triceps brachii* (11.3 points; 62.3 vs. 51.0 points) and the *M. longissimus dorsi* (3.1 points; 66.6 vs. 63.5 points), whereas the grill scored 1.8 points higher (44.3 vs. 46.1 points) for the *M. semimembranosus* [20]. This indicates that the length of the muscle fiber plays an important role in consumer sensory assessment of tenderness for some muscles. If length of muscle fiber impacts consumers sensory scores, then it is likely that consumers in the current study would rate the 70 mm long pulled sample lower than a 6 mm sliced or 10 mm chopped samples.

Muscle differences were noted for tenderness, juiciness, flavor liking, and overall liking. For specific muscles, the PEB056 was scored lower for all consumer sensory scores compared to PEB057 D and PEB057 V. Recent studies have found similar results from consumer sensory testing with PEB056 and PEB057 [6,7]; where one of these studies followed the same protocols as the current study [7]. It is probable that this is associated with muscle fiber type or connective tissue content which has been previously identified to influence sensory evaluations. However, this may also be due to differences in the variable amount of intramuscular fat found in these muscles.

The amount of collagen present in a muscle will affect the sensory outcome of that same muscle, specifically tenderness [21,22]. The cooking method utilized will play a large role in the outcome [20,21,23], given different cooking methods will break down collagen to varying degrees. Results from Jeremiah et al. [21] would suggest that there is no difference between PEB056 and PEB057; however, the briskets were roasted in a convection oven at 177 °C to an internal temperature of 72 °C, possibly not allowing for the full gelatinization of the connective tissue. A study by King et al. [22] found that the *M. triceps brachii*, a muscle known to have a higher collagen content, was more tender when cooked to an internal temperature of 70 °C in a 93 °C oven compared to 260 °C oven.

Belew et al. [24] determined the Warner Bratzler shear force of PEB057 as 3.23 ± 0.20 and PEB056 as 5.12 ± 0.20, identifying that the PEB056 is less tender than PEB057. The same authors measured the Warner Bratzler shear force in 11 different positions within PEB056, noting the varying degrees of tenderness ranging from 4.56 ± 0.31 toward the cranial aspect to 5.54 ± 0.31 toward the middle of the PEB056 [24]. Belew et al. [24] noted that while differences in shear force occurred between some positions in the PEB056, there did not appear to be a trend towards tenderness at one end. This supports the current study’s finding that there was no effect of the position within muscle on consumer sensory evaluations in the PEB056. The additional fat in the PEB057,as compared to the PEB056, may have played a role in the difference in sensory outcomes. Jeremiah [23] determined that there was a higher moisture content (mg/g) and lower fat content in the PEB056 compared with the PEB057.

Briskets were collected from carcasses presenting a wide range of traits; sex, hormone growth promotant treatment, hot standard carcass weight, hump height (i.e., % *Bos indicus*), ossification, MSA marbling, ultimate pH of the *M. longissimus lumborum*, and rib fat depth. This variation was required for the overarching research to assist in the development of a cooking method for low and slow smoked brisket and to determine the effect of the cooking method on briskets of varying degrees of eating quality. The analysis above outlined that sex contributed to variation in eating quality. Previous research has determined that sex plays a small role in eating quality, with females having a slightly lower eating quality than castrated males [1,25]. However, the estimated coefficients in this current study were considerably larger in males for flavor (7.37; *p* < 0.01) and overall liking (10.65, *p* < 0.001); however, there was no difference for tenderness, juiciness, or MQ4 score. Sex was not balanced in the current study, where there was a larger number of grass-fed females with a lower predicted eating quality at grading (i.e., high ossification and low marbling and carcass weight). This may have played a role in the impact of sex and, as such, should be treated with caution. Nonetheless, given the high ossification score, it is interesting to note that there is no difference in tenderness score for sex. The sex effects also remained significant when ossification score and MSA marbling score were included in the model, indicating that the sex effect is not related to physiological maturity or marbling.

Ossification is a measure of physiological maturity and is linked to increased collagen content and reduced tenderness [26,27]. The current study identified that ossification had an impact on tenderness. Given the work by King et al. [22] on cooking temperature and tenderness it may be assumed that the low and slow cooking method has the ability to break down the majority of the collagen and increase the palatability of low-quality cuts, and perhaps even from low-quality carcasses; however, differences still remain. It is suggested that the difference in tenderness seen between the briskets from this range of carcasses is far lower than the difference that would be seen between grilled loin samples from the same carcasses. This is because the low and slow cooking allows for the considerable solubilization of the collagen in the briskets from the more physiologically mature carcasses.

Hormone growth promotants are also known to impact the aging potential, and subsequent tenderness, of beef; however, this only appears relevant in muscles that are able to age post-slaughter [28]. In the current study, HGPs had an effect on the juiciness, flavor liking, overall liking, and MQ4, with tenderness showing a negative trend that was not significant (*p* = 0.07). As with sex, HGP status was not balanced for in this study and tended to be in the lower quality carcasses.

The experiment was designed to explain industry relevant differences between consumers, muscles, and serve methods. The limitations of this experiment are that the mechanisms driving the findings were not evaluated and thus can only be speculated upon. If samples were larger and more funding was available, other traits could have been tested. The measurement of flavor profiles, residual collagen content, shear force, fatty acid profiles, and the intramuscular fat content of the cooked samples would be recommended in future studies.

## 5. Conclusions

The low and slow smoking method was perceived to be acceptable by Australian and USA consumers. While differences occurred between the two countries for tenderness, juiciness, and flavor liking, the MQ4 score was not considered different. The eating quality of the PEB056 was considerably lower than the PEB057, and this identifies a potential for marketing these two muscles independently, particularly for the food service industry. The serve method identified that pulled meat had the lowest eating quality compared to sliced and chopped, which must be considered for consumer satisfaction in a commercial setting and for future data collection on briskets for the MSA model.

## Figures and Tables

**Table 1:** Demographic summary of participants (n = 480) evaluating smoked beef brisket samples in the USA (n = 240) and Australia (n = 240).

Characteristic	Category	AUS, %	USA, %
Age	<20 years old	19.6	7.5
	20–29 years old	18.3	20.0
	30–39 years old	13.8	16.7
	40–49 years old	13.8	26.7
	50–59 years old	26.3	19.2
	60 years old or more	7.9	10.0
	Missing	0.4	
Sex	Male	50	46.3
	Female	50	52.1
	Not Stated/Missing	1.4	1.7
Frequency of Beef Consumption	Daily	2.9	13.8
	4–5 times per week	18.3	31.3
	2–3 times per week	43.3	35.8
	Weekly	23.8	15.0
	Bi-weekly	7.1	2.5
	Monthly	3.8	1.7
	Never	0.8	0.0
Annual Household Income	<$25,000	6.7	3.3
	$25,001–50,000	7.9	12.5
AUD$ for AUS consumers	$50,001–$75,000	10.4	37.1
USD$ for USA consumers	$75,001–$100,000	17.1	27.9
	$100,001–$125,000	18.3	19.2
	$125,001–$150,000	9.6	0
	More than $150,000	22.1	0
	Prefer not to say	7.9	0
Level of Education	Non-high school graduate	3.3	3.3
	High school graduate	35	12.5
	Some college/technical school	24.6	37.1
	College graduate	36.7	27.9
	Postgraduate	0	19.2
	Missing	0.4	0

**Table 2:** Descriptive statistics for additional carcass-level covariates from the 24 carcasses. These covariates (along with sex, feed, and HGP treatment) are typically used as MSA model input.

Trait (Units)	Mean ± Standard Deviation	Min–Max
Carcass weight (kg)	355.2 ± 48.6	279–552
Rib fat (mm)	9.9 ± 4.2	5–22
Ossification score	232.9 ± 134.2	140–590
MSA marbling score (units of 10)	394.6 ± 129.3	260–880
Ultimate pH	5.5 ± 0.1	5.4–5.8
Loin temperature (°C)	5.1 ± 1	2.8–6.7

**Table 3:** Descriptive statistics for primal-level covariates of the 48 primals.

Trait (Units)	Mean ± Standard Deviation	Min–Max
Cold weight (kg)	6.4 ± 1.6	3.1–9.1
Cooking loss (%)	43.7 ± 4.2	35.7–52.6
Cooking time (hours)	6.9 ± 1.5	4.2–10.1

**Table 4:** Estimated coefficients plus marginal and conditional R^2^ for the final sensory models. For each model, variance of the primal-ID and carcass-ID random effect (τ_primal_ and τ_carcass_), variance of the residuals (σ^2^), and the intra-class correlation coefficient (ICC) are also depicted.

Trait (Level)	Tenderness	Juiciness	Flavor Liking	Overall Liking	MQ4
Country tested (USA)	4.84 **	4.44 **	−3.48 *	−0.49	−0.17
Position in muscle (PEB057 D) ^#^	9.96 ***	23.43 ***	5.23 ***	8.97 ***	9.58 ***
Position in muscle (PEB057 V) ^#^	14.66 ***	26.49 ***	8.11 ***	12.38 ***	13.15 ***
Serve (chopped)	4.95 **	4.33 **	3.45 *	5.08 **	4.55 **
Serve (sliced)	6.74 ***	4.34 **	1.00	3.02 *	3.63 **
HGP treatment (yes)	−4.11	−5.75 **	−5.01 *	−5.52 **	−7.12 ***
Log (MSA marbling score)	10.09 *	10.31 **			
Log (ossification score)	−15.76 ***				
Ultimate pH		−23.29 *			
Pre-cook weight		3.05 ***			2.89 ***
Sex (male)			7.37 **	10.65 ***	
Cooking loss percent			0.51 *	0.47	
Intercept	78.73 **	85.65	30.49 **	25.10 *	36.59 ***
Random effects					
τ_primal_	13.45	7.85	8.03	4.78	7.76
τ_carcass_	9.07	1.99	8.36	11.48	8.03
σ^2^	80.46	79.98	59.55	65.16	55.52
ICC	0.22	0.11	0.22	0.20	0.22
Marginal/Conditional R^2^	0.513/0.62	0.7/0.733	0.305/0.455	0.414/0.531	0.457/0.577

* *p* < 0.05; ** *p* < 0.01; *** *p* < 0.001. ^#^ PEB057D is the dorsal portion of *M. pectoralis superficialis* and PEB057V is the ventral portion of *M. pectoralis superficialis.*

**Table 5:** ANOVA table for final sensory models. Type II F-tests are shown, with F-ratios and *p*-values. For reference, two-way interactions between country tested, position in muscle, and serve are included; all are insignificant.

Trait	d.f.	Tenderness		Juiciness		Flavor Liking		Overall Liking		MQ4	
		F	*p*-Value	F	*p*-Value	F	*p*-Value	F	*p*-Value	F	*p*-Value
Country tested	1	7.42	0.01	7.79	0.01	4.79	0.04	0.11	0.74	0.01	0.90
Position in muscle	2	46.15	<0.001	178.92	<0.001	18.01	<0.001	40.96	<0.001	54.93	<0.001
Serve method	2	8.78	<0.001	4.54	0.01	2.97	0.05	5.59	<0.01	5.88	<0.01
HGP treatment	1	3.77	0.07	9.01	<0.01	6.27	0.02	6.97	0.01	13.21	<0.01
Log (MSA marbling)	1	5.36	0.03	8.76	<0.01						
Log (ossification score)	1	38.94	<0.001								
Ultimate pH	1			5.23	0.03						
Cold weight	1			25.43	<0.001					22.15	<0.001
Sex	1					8.17	<0.01	16.62	<0.001		
Cooking loss percent	1					3.57	0.07	3.00	0.09		
Country tested/ position in muscle	2	1.89	0.16	1.50	0.23	0.23	0.80	1.16	0.32	1.24	0.29
Country tested/ serve method	2	2.36	0.10	2.60	0.08	1.12	0.33	1.50	0.23	2.51	0.08
Position in muscle/ serve method	4	0.16	0.96	0.38	0.82	0.32	0.86	0.04	0.996	0.13	0.97

**Table 6:** Estimated marginal means (EMMs) ± standard error and their 95% confidence intervals for sensory scores of briskets tasted in Australia and USA. For each sensory score, the contrast (USA–AUS) was estimated and tested using a *t*-test.

Sensory	Country	Emmeans ± SE	95% CI	Estimated (USA-AUS) Contrast ± SE	*p*-Value
Tenderness	Australia	63.4 ± 1.42	(60.5, 66.3)	4.84 ± 1.70	<0.001
	USA	68.2 ± 1.42	(65.4, 71.1)		
Juiciness	Australia	55.3 ± 1.18	(52.9, 57.6)	4.44 ± 1.55	<0.01
	USA	59.7 ± 1.18	(57.3, 62.1)		
Flavor Liking	Australia	60.1 ± 1.47	(57.1, 63)	−3.48 ± 1.58	0.037
	USA	56.6 ± 1.34	(53.9, 59.3)		
Overall Liking	Australia	58.0 ± 1.51	(54.9, 61.1)	−0.49 ± 1.54	0.752
	USA	57.5 ± 1.38	(54.7, 60.3)		
MQ4	Australia	62.0 ± 1.15	(59.6, 64.3)	−0.17 ± 1.36	0.881
	USA	61.8 ± 1.15	(59.5, 64.1)		

## Data Availability

The original contributions presented in the study are included in the article, further inquiries can be directed to the corresponding author.
